# Editorial: Connected Health: Status and Trends

**DOI:** 10.3389/fdgth.2021.762270

**Published:** 2021-10-20

**Authors:** Constantinos S. Pattichis, Andreas S. Panayides, Chris Nugent

**Affiliations:** ^1^Department of Computer Science and Biomedical Engineering Research Centre, University of Cyprus, Nicosia, Cyprus; ^2^CYENS Center of Excellence, Nicosia, Cyprus; ^3^School of Computing, Ulster University, Coleraine, United Kingdom

**Keywords:** connected health, digital health, eHealth, mHealth, telemedicine

## Introduction

Recent advances in information and communication technologies prescribe an emerging paradigm in the delivery of advanced healthcare services named connected health. The scope of this special issue, is fully aligned with the Frontiers in Digital Health, section on Connected Health (1). The aim of this special issue is to present selected papers describing recent advances in connected health systems, platforms and solutions targeted to the development of efficient and effective interventions toward the provision of healthcare services for the benefit of the citizen. Topics covered include, sensing and Internet of Things (IoT) in healthcare systems, personalized and well-being systems, early diagnostics and clinical diagnostics, electronic health records and integrated care, and security and data protection. The manuscripts cover the aforementioned technologies toward the development of citizen-centric personalized eHealth, mHealth, pHealth and uHealth solutions, in addition to telemedicine systems.

## A Brief Overview of the Papers in the Special Issue

The papers in this special issue can be grouped under the following three categories that are briefly presented in this section: patient engagement via sensing, monitoring and coaching systems; emerging disease biomarkers; and eHealth eco system enabling technologies.

### Patient Engagement *via* Sensing, Monitoring, and Coaching Systems

The first paper by Androutsou et al. presents a mobile application that is part of a multifactorial intervention to support the aging with balance disorders. This application enables the users to self-evaluate their activity and progress, to communicate with each other and to engage with the system with undiminished interest for a long period of time. The application is expected to undergo an evaluation in four pilot studies with 160 participants.

The second paper by Schütz et al. introduces a calibration framework of passive infrared motion sensors that covers the quantification of simultaneously acquired data from wearable accelerometers and use the data to find a suitable correlation between in-home and out-of-home physical activity. The proposed framework was evaluated by 20 community-dwelling older adults for at least 60 days. It was found that passive infrared based wireless sensor systems can be calibrated to give largely better estimates of older adults' daily physical activity using data over 7–14 days only.

The paper by Ntracha et al. introduces smartphone interaction behavioral data, unobtrusively captured as an aid in the screening and monitoring of Mild Cognitive Impairment (MCI). Digital biomarkers drawn from Fine Motor Impairment and Spontaneous Written Speech related data analysis were investigated on matched groups of 11 MCI patients and 12 healthy controls, over a time span of 6 months. The results demonstrate the potential of the proposed biomarkers to detect early stages of cognitive decline.

The fifth paper in this subsection by Tsiouris et al. reviews 41 papers that were published in the last decade covering the topic of virtual coaching systems targeted to enhance healthcare interventions. The findings suggest that home coaching systems were mainly focused on physical activity and a healthier living based on IoT devices and sensor monitoring. The devices that were used were mostly activity trackers, pedometers and heart rate monitoring. It is documented that real-time performance evaluation and personalized feedback was found to be rather lacking. Future trends in home coaching systems target to close the loop with real-time automated performance evaluations, monitoring, feedback, and more advanced interventions.

### Emerging Disease Biomarkers

Juvenile idiopathic arthritis (JIA) is the most common rheumatic disease of childhood. It is very difficult to assess JIA due to its highly variable presentation and the documentation of few reliable biomarkers to assess it. In the paper by Whittingslow et al. the joint acoustic emissions (JAEs) from the knees of children with JIA are quantified and proposed as a new biomarker for the non-invasive assessment of the disease. JAEs from 25 patients with JIA were evaluated with very promising findings suggesting the use of the proposed technique in clinical practice.

Stanitsas et al. propose a system that provides useful features that describe malignant regions in cancerous tissue. A Covariance-Kernel based patch descriptor is introduced that has the capability of describing tissue carcinoma. The proposed methodology was evaluated on a breast cancer dataset with very promising results.

### Ehealth Eco System Enabling Technologies

The paper by Martin et al. proposes an immersive environment to track behaviors relevant to neuropsychiatric symptomatology. The proposed framework facilitates connected tele-psychiatry, providing quantitative effective assessment, and thus overcoming subjective symptomatology analysis that is the current practice.

The study by Christoforou et al. provides an overview of assistive robotics in nursing, summarizing the benefits of the proposed solutions in clinical practice. Moreover, the paper presents the end-users' perspective and identifies challenges, limitations and future directions of the robotic solutions.

The paper by Smith et al. reports the 15 years' experience (2000–2016) of the Queensland Telepaediatric Service (QTS) that was established in Brisbane, Australia. QTS was developed to support telehealth services in remote locations. A total of 23,054 telehealth consultations were delivered for 37 pediatric clinical specialties. The most common services covered the following: child and youth mental health, neurology, burns care, surgery, and ear nose and throat services. Most of the health services involved hospital video consultations for the delivery of the services.

A citizen-centric framework which enables EHR system integration with biobanks is proposed in the paper by Antoniades et al. The proposed framework enables retrospective lifelong prospective longitudinal studies, adhering strictly to legal and ethical requirements. Citizens would benefit through the proposed system, supporting them to make informed decisions and exercising their rights related to the use of their data.

Finally, the last paper in this special issue by Spanakis et al. aims to provide a framework of new technologies for the implementation of a secure information sharing platform for health data. The methodology presented in this paper documents strategies for information sharing, high-level requirements for the transfer of data between health-care organizations, technologies to support secure interconnectivity and trust, standards, guidelines, and interoperability specifications. Moreover, the use of cloud computing in sharing health data is covered, including also more advanced solutions such as block chain.

## Concluding Remarks

The coronavirus disease (COVID-19) pandemic increases the need and demand for ongoing connected health and digital health interventions, solutions and tools (2). Virtual tele consultations have become the preferred mode of operation, rapidly replacing traditional face-to-face consultations given the increased risk of infection (3). Furthermore, in a recently published paper by Ding et al. (4), the following connected health enabling technologies and systems for handling the COVID-19 crisis are documented: “(i) wearable devices for monitoring populations at risk and those in quarantine that are needed for evaluating the health status of caregivers and for triggering triage processes for admission to hospitals; (ii) unobtrusive sensing systems for detecting the disease and for monitoring patients with mild symptoms whose clinical situation could suddenly worsen and trigger also admission to hospitals; and (iii) telehealth technologies for the remote monitoring and diagnosis of COVID-19 and related diseases.”

The papers presented in this special issue provide connected health solutions and tools for minimizing the thread of the pandemic. Moreover, the papers presented provide a snapshot of recent advances in connected health, hoping to drive and accelerate the development, translation, and application of connected health into clinical practice toward improved disease management and treatment at the point of care, reduced hospitalization, offering a better quality of life to the citizen, at reduced cost. There is no doubt that connected health and digital health will continue to transform healthcare practice and services for the benefit of the citizen.

## Author Contributions

All authors listed have made a substantial, direct and intellectual contribution to the work, and approved it for publication.

## Funding

CP acknowledges partial funding from the CYENS Centre of Excellence via the European Union's Horizon 2020 research and innovation programme under grant agreement No. 739578 and the funding received from the Government of the Republic of Cyprus through the Deputy Ministry of Research, Innovation and Digital Policy. Invest Northern Ireland is acknowledged for partially supporting this work under the Competence Centre Programs Grant RD0513853 Connected Health Innovation Centre.

## Conflict of Interest

The authors declare that the research was conducted in the absence of any commercial or financial relationships that could be construed as a potential conflict of interest.

## Publisher's Note

All claims expressed in this article are solely those of the authors and do not necessarily represent those of their affiliated organizations, or those of the publisher, the editors and the reviewers. Any product that may be evaluated in this article, or claim that may be made by its manufacturer, is not guaranteed or endorsed by the publisher.
